# Effects of Feed Restriction on Growth Performance, Nutrient Utilisation, Biochemical Parameters, and the Caecum Microbiota and Metabolites in Rabbits

**DOI:** 10.3390/ani15060842

**Published:** 2025-03-14

**Authors:** Qi Lu, Jixiao Qin, Shuanglong Xie, Rui Chen, Xu Wang, Yiqing Xu, Yiming Ban, Chengcheng Gao, Peiyao Li, Di Zhou, Xingzhou Tian

**Affiliations:** 1 Laboratory of Animal Genetics, Breeding and Reproduction in the Plateau Mountainous Region, Ministry of Education, College of Animal Science, Guizhou University, Guiyang 550025, China; luqi2556728@163.com (Q.L.); zrz2942037436@163.com (J.Q.); xsljty@163.com (S.X.); chenruisc@163.com (R.C.); wx12345678888@163.com (X.W.); tacool1001@163.com (Y.X.); 18886411543@163.com (Y.B.); 16684967520@163.com (C.G.); 16987129454@163.com (P.L.); 2 Guizhou Testing Centre for Livestock and Poultry Germplasm, Guiyang 550018, China

**Keywords:** feed restriction, nutrient utilisation, antioxidant, microbiome, metabolome, rabbits

## Abstract

The main objective of this research was to observe the effects of feed restriction on caecum microbiota and metabolites in rabbits. Forty-eight male 8-week-old rabbits with similar body weights (1872.11 ± 180.85 g) were randomly assigned to two treatments according to completely randomized design: (1) the control group received *ad libitum* access to feed (AL), and (2) the treatment received 80% of the feed consumed by the control (FR). The results showed that FR did not differ (*p* > 0.05) for average daily weight gain or feed conversion ratio between the two groups. FR treatment led to a significant increase (*p* < 0.05) in acid detergent fibre apparent faecal digestibility, nitrogen digestibility and retention, and gross energy digestibility and retention. The FR treatment showed significantly (*p* < 0.05) lower blood triglycerides, creatinine, high-density lipoprotein cholesterol, malondialdehyde, and hydroxyl free radicals but significantly (*p* < 0.05) greater total antioxidant capacity and superoxide dismutase. The FR group presented greater (*p* < 0.05) *Firmicutes* and *Ruminococcus* abundances but a lower (*p* < 0.05) *Akkermansiaceae* abundance in the caecal content. Moreover, 222 differentiated metabolites were identified, and beta-alanine metabolism was the top enriched pathway. Collectively, FR can improve nutrient utilisation, lipid metabolism, antioxidant activity, caecum microbiota, and metabolites in rabbits.

## Simple Summary

Feeding restriction refers to artificially controlling the amount of feed consumed by animals so that the quality of the nutrients consumed is lower than the animal’s demand level, which can effectively improve the body’s immune function and disease resistance. Feed restriction has been used to improve body health in growing rabbits. In addition, meat rabbits are monogastric herbivores with developed caeca and a strong ability to decompose and digest nutrients. However, the effects of feed restriction on the caecum microbiota and metabolites in rabbits remain unclear. Thus, in the present study, we investigated the effects of feed restriction on the growth performance, nutrient utilisation, lipid metabolism, antioxidant activity, caecal microbiota, and metabolites of rabbits. Our research results provide a theoretical reference for the development of healthy feeding restriction standards for meat rabbits.

## 1. Introduction

Mild feed restriction (FR) is beneficial for the health of animals, as it can improve digestive system health and reduce the incidence of animal epidemics [1,2]. For example, Halai et al. [3] reported that FR could improve health in male mice by increasing the mean lifespan and influencing biochemical parameters. Similarly, rats fed a 30% restriction diet showed regulated disordered gut microbiota and inhibited metabolic endotoxaemia and inflammation [4]. Similar findings have also been found in rabbits. Gidenne et al. [5] showed that 80% FR did not affect carcass traits, whereas it reduced the feed conversion ratio (FCR) and the mortality of young rabbits. In addition, Martignon et al. [6] reported that 75% FR significantly increased nutrient digestibility and decreased mortality in weaned rabbits. Thus, FR may improve body health by regulating antioxidant capacity, lipid metabolism, and immune function in animals [7,8].

FR has been used to improve the performance of rabbits, pigs, lambs, chickens, and turkey [9]. Previous studies have shown that FR affects the gastrointestinal tract microbiota and metabolites [10,11]. FR can significantly modulate the microbiota of the caecum and increase immune and antioxidant functions in rats [4]. Artdita et al. [12] showed that 80% FR could decrease excessive fat without affecting the egg production rate and upregulate lipid, carbohydrate, amino acid, and nucleic acid pathways in the caecum in late-stage laying hens, as shown by a Kyoto Encyclopedia of Genes and Genomes (KEGG) assay. In addition, Makovicky et al. [13] indicated that FR could increase mean villus height and the length of the small intestine in weaned *Hyplus* rabbits. Similarly, FR increased digestible area in the small and large intestines (consisting of villus height and crypt depth) in growing rabbits [14]. Hence, FR might affect the gut microbiota and metabolism and may be a healthy dietary strategy for improving animal health [15,16].

The rabbit caecum represents a structurally highly developed organ where feed can be fermented, which is crucial for digestion and nutrient absorption [17]. Previous studies with rabbits have indicated that *ad libitum* feeding might have a negative effect on digestive disorders and that FR preserves digestive health and thus reduces mortality and morbidity rates [18,19,20]. Furthermore, 80% FR has been widely applied in growing rabbit breeding systems to improve body health [5,21]. For example, Crespo et al. [22] found that 80% FR did not affect the mortality and growth rate but improved nitrogen and energy utilization in New Zealand × Californian crossbred hybrid rabbits. Moreover, our previous study did show that 80% FR could enhance muscle antioxidant activity and meat quality in growing rabbits [23]. However, previous studies have mainly investigated the effects of FR on the growth performance, meat quality, mortality, and apparent faecal digestibility [6,22,23]; the effects of FR on the caecal microbiota and metabolites in rabbits remain unclear. We hypothesize that mild FR enhances nutrient utilisation, improves antioxidant activity, and alters caecal microbiota composition by increasing beneficial bacteria and modifying metabolic pathways in growing rabbits. Accordingly, this study aimed to investigate the effects of FR on the growth performance, nutrient utilisation, lipid metabolism, antioxidant activity, and caecal microbiota and metabolites of rabbits.

## 2. Materials and Methods

### 2.1. Animals and Experimental Design

All animal procedures were reviewed and approved by the Guizhou University Animal Ethics Committee (No. EAE-GZU-2024-E028). In general, meat rabbits are slaughtered at age 3–4 months, and our previous study showed that 80% FR resulted in improved meat quality in rabbits [23]. Therefore, forty-eight male, 8-week-old pure New Zealand white rabbits with similar body weights (1872.11 ± 180.85 g; mean ± standard deviation) were randomly assigned to two equal treatment groups in a completely randomized design. The control group received *ad libitum* access to feed (AL), and the treatment group received 80% of the feed consumed by the control (FR). The restriction programme was applied by giving a daily meal starting at 8 weeks. Before the start of the experimental period, a pretest was conducted to calculate the feed intake of 24 randomly fed rabbits. This provided the feed consumption for both groups during the restricted feeding period. Moreover, daily feed intake was calculated, and the amount of rabbit feed was adjusted weekly. The diets were fed in equal amounts twice daily at 08:30 and 17:30. The feeding trial lasted for 7 weeks and included a 1-week pretrial period and a 6-week formal period. The feed of the rabbits in the AL group was gradually reduced to 80% over 4 d, and the rabbits were allowed to acclimate to 80% for 3 d before beginning the experimental period. Each treatment consisted of 12 replicates (*n* = 12) with 2 rabbits each (2 rabbits per cage). The rabbits were placed in pairs in cages (160 × 70 × 195 cm). All rabbits had free access to water during the whole feeding period. The experimental diet was manufactured and pelleted with a 4 mm millstone by a granule presser at one time (Jixiang Animal Husbandry Machinery Co., Ltd., Zhengzhou, China) using one batch of raw materials. The nutritional requirements were followed according to the Chinese standard (NY/T 4049-2021) [24], and the ingredients of the basal diet are shown in Table 1.

The dry matter (DM; method 934.01), crude protein (CP; method 988.05), and ash (method 942.05) contents were analysed according to the AOAC [25]. The neutral detergent fibre (NDF) and acid detergent fibre (ADF) contents were analysed according to the methods of Van Soest et al. [26]. The gross energy (GE) was analysed using an adiabatic oxygen bomb calorimeter (WGR-WR3, Changsha Bente Instrument Co., Ltd., Changsha, China). The chemical composition of the basal diet is shown in Table 2.

### 2.2. Growth Performance and Digestibility of Feed Nutrients

The feed weight was measured every day to monitor the dry matter intake (DMI). The animal body weight was weighed on the first day (initial weight) and last day (final weight), and the average daily weight gain (ADG) was calculated. The FCR was calculated by dividing the DMI by the ADG. In addition, no rabbits died throughout the entire experimental period.

The digestion and metabolism experiment was carried out at 100–105-day-old rabbits. Briefly, faecal and urine samples were collected during the last 5 d using the total faecal and urinary collection method. The faeces was divided into two parts: the first part was stored directly in a −20 °C refrigerator, and the second part was added to 20% H_2_SO_4_ to determine the N content. At the end of the experiment, the faecal sample was dried under vacuum at 60 °C for 72 h, ground, and passed through a 1 mm sieve for chemical composition analysis. The urine was added to 20% H_2_SO_4_ to maintain the pH at lower than 3 and kept at −20 °C for N and GE analysis.

### 2.3. Plasma Parameters

The plasma samples were collected from all tested rabbits on the last day of the experiment. Before slaughter, blood was collected from the heart using a vacuum blood collection tube (Kangweishi Medical Technology Co., Ltd., Shijiazhuang, China). Then, the blood was centrifuged for 10 min at 4000× *g* (TD4, Shanghai Lu Xiangyi Centrifuge Instrument Co., Ltd., Shanghai, China), and the plasma was stored at −80 °C for later testing. The following plasma parameters were detected: (1) biochemical parameters: glucose (Glu), total protein (TP), and albumin (Alb); (2) lipid metabolism parameters: triglyceride (TG), creatinine (Cr), total cholesterol (TCH), low-density lipoprotein cholesterol (LDL-C), and high-density lipoprotein cholesterol (HDL-C); and (3) antioxidant activity parameters: total antioxidant capacity (T-AOC), superoxide dismutase (SOD), glutathione peroxidase (GSH-Px), catalase (CAT), 2,2-diphenyl-1-picrylhydrazyl (DPPH), free radical scavenging capacity (DPPH scavenging activity), malondialdehyde (MDA), hydroxyl free radical (·OH), and superoxide anion (O_2_). All kits were obtained from the Nanjing Jiancheng Bioengineering Institute (Nanjing, China).

### 2.4. Caecal Microbiota Analysis

After the slaughter, 24 caecum samples were immediately collected from each group (2 rabbits/cage), and the caecum samples from 2 rabbits per cage were pooled. Then, the microbiome and metabolome were analysed. The DNA was extracted from the caecal samples by the E.Z.N.A.^®^ soil DNA Kit (Omega Bio-tek, Norcross, GA, USA). The DNA extract was examined on 1% agarose gel, and the DNA concentration and purity were detected using a NanoDrop 2000 UV–vis spectrophotometer. The hypervariable region V3–V4 of the bacterial 16S rRNA gene was amplified using the primer pair 338F (5′-ACTCCTACGGGAGGCAGCAG-3′) and 806R (5′-GGACTACHVGGGTWTCTAAT-3′) by an ABI GeneAmp^®^ 9700 PCR thermocycler (Foster City, CA, USA).

Purified amplicons were pooled in equimolar amounts and paired-end sequenced on an Illumina MiSeq PE300 platform/NovaSeq PE250 platform (Illumina, San Diego, CA, USA). The raw 16S rRNA gene sequencing reads were demultiplexed, quality filtered by fastp version 0.20.0, and merged by FLASH version 1.2.7. Operational taxonomic unit (OTU) with 97% similarity cut-off was clustered by the UPARSE version 7.1, and chimeric sequence was identified and removed. The taxonomy of each OTU representative sequence was analysed using the RDP Classifier version 2.2 against the 16S rRNA database (e.g., SILVA v138) with a confidence threshold of 0.7. The raw reads were deposited into the NCBI Sequence Read Archive (SRA) database (Accession Number: PRJNA1222279).

### 2.5. Caecal Metabolomic Analysis

A total of 12 caecum samples (2 rabbits per cage were pooled) were analysed for their metabolome. A 100 g caecal sample was added to 400 μL of solution (acetonitrile/methanol = 1:1 (*v*:*v*)) containing 0.02 mg/mL internal standard (L-2-chlorophenylalanine) to extract metabolites. The sample was mixed by vortexing for 30 s and then sonicated at 5 °C for 30 min with 40 kHz. Next, the supernatant was removed after centrifugation at 13,000× *g* for 15 min at 4 °C and then was blown dry under nitrogen. The sample was then resolubilized with 100 µL of solution (acetonitrile/water = 1:1) and extracted by low-temperature ultrasonication for 5 min at 5 °C with 40 kHz. The supernatant was collected after centrifugation at 13,000× *g* for 10 min at 4 °C for liquid chromatography–mass spectrometry (LC-MS/MS) analysis.

The data matrix obtained by searching the database was uploaded to the Majorbio cloud platform (https://cloud.majorbio.com, accessed on 25 October 2023). The orthogonal least partial squares–discriminant analysis (OPLS-DA) was analysed by the R package “ropls” (version 1.6.2), and the stability of the model was evaluated by the 7-cycle interactive validation. Differentially abundant metabolites were mapped to their biochemical pathways by metabolic enrichment and pathway analysis based on the KEGG database (http://www.genome.jp/kegg/, accessed on 25 October 2024).

### 2.6. Statistical Analysis

One cage (2 rabbits per cage) was considered an experimental unit in this study. The sample sizes were analysed by Statistical Analysis System 9.1.3 (SAS Institute, Cary, NC, USA); twelve replicates (*n* = 12) were shown to obtain a power of 0.80 and a 0.05 significance level, according to our previous study [23]. Data on growth performance, nutrient digestibility, and plasma parameters were analysed by Student’s *t*-test using SAS 9.1.3 software. The abundances of the microbiota were analysed by the Kruskal–Wallis H test. The metabolites with variable importance in the projection (VIP) > 1 and *p* < 0.05 were detected to be significantly differentially abundant metabolites obtained by the OPLS-DA model and the *p*-value generated by the Student’s *t*-test.

## 3. Results

### 3.1. Dry Matter Intake, Growth Performance, and Apparent Faecal Digestibility

The DMI value in the FR treatment group was significantly lower (*p* < 0.05) than that in the AL treatment (Table 3).

In contrast, no significant differences (*p* > 0.05) were observed for the initial weight, final weight, ADG, or FCR values between the two treatments. The apparent faecal digestibilities of DM, NDF, and ash did not differ (*p* > 0.05) between the two treatments. In addition, compared with the AL treatment, the FR treatment led to a significant increase (*p* < 0.05) in ADF apparent faecal digestibility (increased 12.44%).

### 3.2. Nitrogen Utilisation

Compared with the AL group, the FR group presented lower (*p* < 0.05) levels of N intake, N excretion in faeces, N excretion in urine, and total N excretion (Table 4). However, FR treatment showed significantly (*p* < 0.05) higher N digestibility and N retention contents relative to the AL treatment.

### 3.3. Energy Utilisation

Compared with the AL group, the FR treatment group presented lower (*p* < 0.05) concentrations of GE intake, GE excretion in faeces, GE excretion in urine, and total GE excretion (Table 5). In contrast, the FR treatment had significantly (*p* < 0.05) higher GE digestibility and GE retention levels compared to the AL group.

### 3.4. Plasma Biochemical, Lipid Metabolism, and Antioxidant Activity

There were no significant differences (*p* > 0.05) detected for the plasma Glu, TP, Alb, TCH, LDL-C, GSH-Px, CAT, and DPPH scavenging activities or O_2_· concentrations between the two groups (Table 6). The FR treatment showed significantly (*p* < 0.05) lower values of TG, Cr, HDL-C, MDA, and ·OH in plasma compared with the AL group. In contrast, the FR treatment had significantly (*p* < 0.05) higher T-AOC and SOD activities relative to the AL group.

### 3.5. Caecal Microbiota

**Figure 1:**
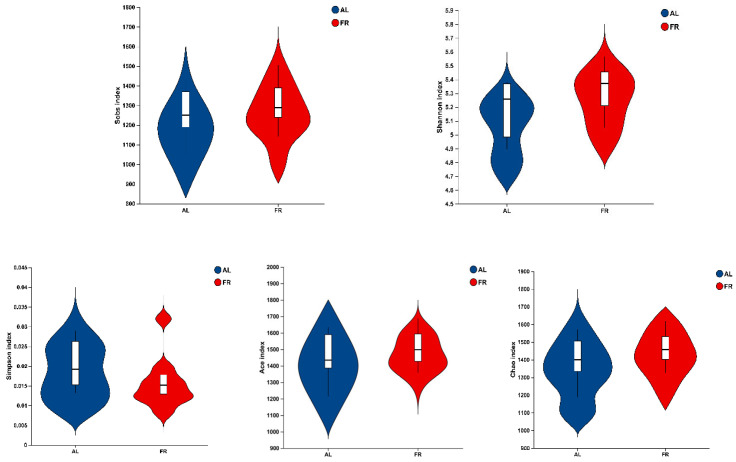
Analysis of α diversity index in caecal content between AL and FR treatments in rabbits. AL, rabbit was fed *ad libitum* diet; FR, rabbit was fed 80% *ad libitum* diet.

A total of 1,590,531 raw reads were determined in this study among the 24 samples. A total of 1,514,566 effective tags were identified by eliminating low-quality sequences. The effective number of bases was 618,406,083 bp, and the average length was 408 nt from 24 samples. In terms of α diversity, there was no (*p* > 0.05) significant difference in the Sobs, Shannon, Simpson, Ace, or Chao1 index between the AL and FR treatments (Figure 1).

**Figure 2:**
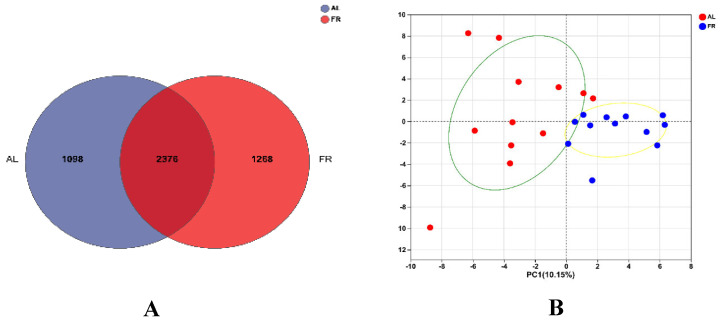
Effect of FR on the caecal bacterial communities in rabbit. (**A**) Comparison of Venn diagram among the two treatments; (**B**) principal component analysis of bacterial communities. AL, rabbit was fed *ad libitum* diet; FR, rabbit was fed 80% *ad libitum* diet.

For the OTU cluster analysis, a total of 4709 OTUs were obtained, which were classified into 12 phyla, 20 classes, 45 orders, 77 families, 171 genera, and 349 species. Specifically, 2376 OTUs were shared between the two groups (Figure 2A), 1098 were exclusive to the AL group, and 1268 were exclusive to the FR group. Principal component analysis showed that the caecal microbiota was different between the two treatments (Figure 2B).

**Figure 3:**
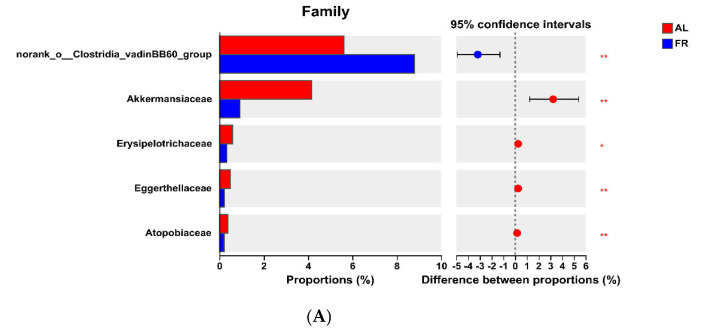

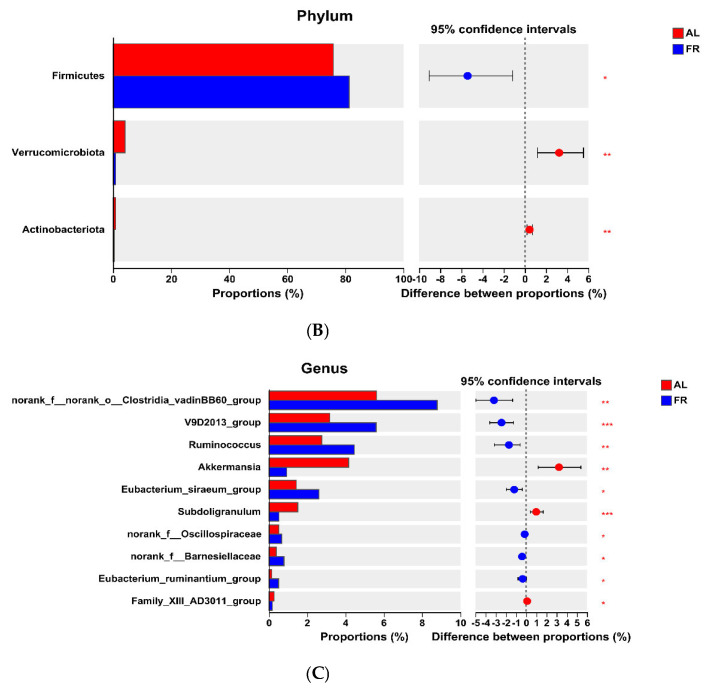
Significant differences of caecal microbiota analysis. (**A**) Phylum level. (**B**) Family level. (**C**) Genus level. * *p* < 0.05, ** *p* < 0.01, and *** *p* < 0.001 mean the significant difference between groups. AL, rabbit was fed *ad libitum* diet; FR, rabbit was fed 80% *ad libitum* diet.

At the phylum level, the FR group presented greater (*p* < 0.05) *Firmicutes* abundance in the caecal content but lower (*p* < 0.05) *Verrucomicrobiota* and *Actinobacteriota* abundances than the AL group did (Figure 3A). At the family level, the FR treatment led to a greater (*p* < 0.05) abundance in the caecal content of norank_o__*Clostridia*_vadinBB60_group and lower (*p* < 0.05) abundances of *Akkermansiaceae*, *Erysipelotrichaceae*, *Eggerthellaceae*, and *Atopobiaceae* compared with the AL group (Figure 3B). At the genus level, FR treatment resulted in greater (*p* < 0.05) abundances in the caecal content of norank_f__norank_o__*Clostridia*_vadinBB60_group, V9D2013_group, *Ruminococcus*, *Eubacterium*_*siraeum*_group, norank_f__*Oscillospiraceae*, norank_f__*Barnesiellaceae*, and *Eubacterium*_*ruminantium*_group but lower (*p* < 0.05) abundances of *Akkermansia*, *Subdoligranulum*, and Family_XIII_AD3011_group compared with the AL group (Figure 3C).

### 3.6. Caecal Metabolome

**Figure 4:**
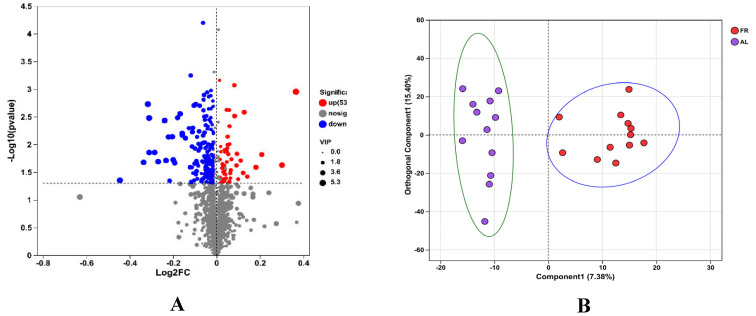
Differential caecal metabolites in this study. (**A**) Volcano plot. (**B**) Orthogonal projections to latent structure discrimination analysis. AL, rabbit was fed *ad libitum* diet; FR, rabbit was fed 80% *ad libitum* diet.

The metabolome of the caecal content samples was determined using LC-MS/MS, and 2114 metabolites were detected in the two groups (Figure 4A).

With respect to the fold change (FC) and VIP of metabolites in the FR or AL groups, 222 differentiated metabolites were identified (positive and negative ions). Among the 222 metabolites, 53 metabolites were classified as upregulated, and 169 were downregulated (Figure 4A).

The OPLS-DA results showed that the metabolites of the FR treatment could be completely separated from those of the AL group, indicating that FR treatment could change the caecal metabolites (Figure 4B).

**Figure 5:**
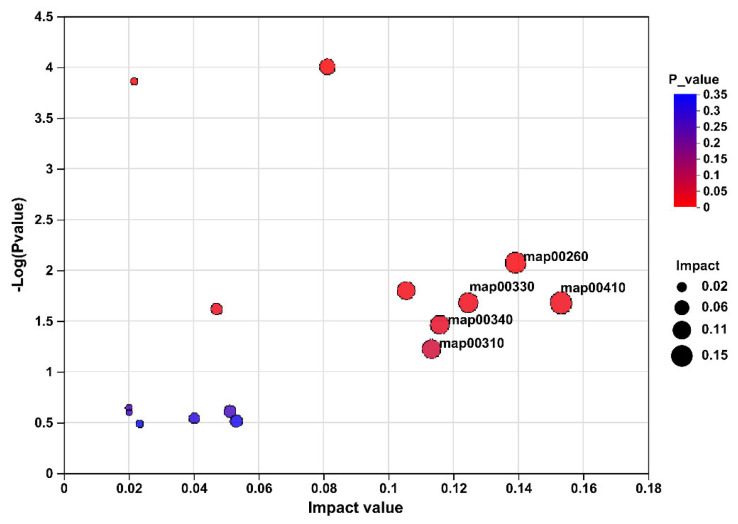
The pathway analysis of caecal content differentially expressed metabolites based on the comparison of rabbits in AL and FR. map00410, beta-alanine metabolism; map00260, glycine, serine, and threonine metabolism; map00330, arginine and proline metabolism; map00340, histidine metabolism; map00310, lysine degradation. AL, rabbit was fed *ad libitum* diet; FR, rabbit was fed 80% *ad libitum* diet.

To further identify the target metabolites modulated by FR, metabolic pathway analysis of these 222 differentially abundant metabolites revealed enrichment of pathways, the top five of which included beta-alanine metabolism (pathway id map00410), glycine, serine and threonine metabolism (pathway id map00260), arginine and proline metabolism (pathway id map00330), histidine metabolism (pathway id map00340), and lysine degradation (pathway id map00310; Figure 5).

## 4. Discussion

The effect of FR on growth performance relies on the severity and duration of FR and the age of the growing rabbits [27]. The restriction of feed intake and feeding time for meat rabbits is related to improvements in the health status, digestion, and feed conversion rate of growing rabbits [28,29]. Zhuang et al. [30] reported that 85% FR did not affect the final weight, ADG, or FCR of growing weanling rabbits. FR might improve the small intestine and caecum health and affect the caecum contents and thus might contribute to the animals’ growth [9]. In addition, FR reduced the pathogenic microbiota population and improved the beneficial microbiota population in the caecal content of growing rabbits [31,32]. In the present study, we found that FR did not show different growth performance parameters, perhaps because FR promoted nutrient utilisation and regulated caecal microbiota. This phenomenon was also validated in terms of nutrient digestibility, nitrogen, and energy utilisation (Table 3, Table 4 and Table 5). Consistent with our findings, Romero et al. [33] indicated that 85% FR did not affect the growth performance or FCR values of growing rabbits.

The feed utilisation rate is an indicator that reflects the degree to which feed is decomposed and absorbed in the digestive tract [34]. Nutrias that received 75% FR showed improvements in the length of the caecum and the small intestine [9]. Martignon et al. [6] suggested that FR increased organic matter, GE, CP, NDF, ADF, and hemicellulose digestibilities in New Zealand white × Californian rabbits. Thus, we found that FR increased nutrient digestibility and utilisation in growing rabbits, possibly because FR promotes caecal development and thus improves the digestion and utilisation efficiency of nutrients. Another possible reason might be that FR prolongs feed retention in the gastrointestinal tract, stimulates digestive enzymes, and improves mucosal absorption in feed-restricted rabbits [21]. Consistent with our observations, Martignon et al. [6] reported that FR increased CP, GE, and fibre digestibilities in growing rabbits. Similarly, Gidenne et al. [35] reported that FR improved the N balance, with 40% less total (faeces + urine) N excretion in restricted rabbits. Combes et al. [31] reported that the microbiota is a beneficial source of digestive health biomarkers. Thus, another possible reason might be that FR increases the abundances in the caecal content of some digestive microbiota, such as *Firmicutes* and *Ruminococcus* (Figure 3), in rabbits.

FR can improve lipid metabolism and promote the bodily health of rabbits [36]. This might be because FR can significantly alter the liver lipid deposition-related gene expression and subsequently regulate lipid metabolism in animals [37]. For example, restricted feeding improved deranged lipid profiles (TCH, TG, HDL-C, and LDL-C) in rats [38]. In addition, Liu et al. [39] reported that calorie restriction decreased blood TCH, TG, LDL-C, and HDL-C concentrations in castrated male pigs. Similarly, in the present study, FR resulted in reduced plasma TG, Cr, and HDL-C values in rabbits, suggesting its anti-hyperlipidaemic effect. This was probably because FR downregulates key lipogenic genes (e.g., sterol regulatory element-binding transcription factor 1 and peroxisome proliferator-activated receptor) in animals [40]. Consistent with our findings, Chen et al. [41] reported that FR decreased blood TCH and HDL-C values in broiler chickens.

The T-AOC can reflect the body’s compensation level for external environmental factors and free radical metabolism in animals [42]. SOD is an important antioxidant enzyme that can eliminate the superoxide anion radicals generated during biological oxidation processes and enhance antioxidant capacity in animals [43]. MDA is an oxidative end product, and the level of MDA in the body reflects the degree of peroxidation damage in animals [44]. A previous study showed that FR can increase innate immunity, which might enhance antioxidant activity in animals [45]. For example, Chen et al. [41] reported that FR increased the serum SOD content and reduced the MDA value in broiler chickens. Thus, we found that FR enhanced T-AOC and SOD activities but decreased the MDA and ·OH contents, possibly because FR reduced mitochondrial free radical production and alleviated oxidative damage in animals [46]. Consistent with our findings, Lu et al. [23] reported that FR might increase antioxidant potential in growing rabbits by increasing the muscle SOD activity and DPPH free radical scavenging activity and decreasing the MDA content. Similarly, Zhuang et al. [30] reported that 70% FR increased SOD and reduced MDA levels in the jejunum of New Zealand rabbits at 70 d.

The caecum of meat rabbits is highly developed and contains many beneficial microorganisms that can fully decompose the partially digested chyme in the small intestine and maintain a normal intestinal microbiota, thus maintaining the health of the body [47]. A previous study revealed that 80% FR can improve lipid metabolism and meat quality by altering the structure of the caecal microbial community in animals [48]. In addition, the caecum of meat rabbits contains abundant microorganisms, which can secrete cellulases to degrade plant cellulose in the caecum [49]. Specifically, *Firmicutes*, an important component of the gut microbiota, are involved in the digestion and absorption of feed and in the degradation of cellulose [50]. Therefore, the current results showed that FR increased caecal *Firmicutes* abundance in growing rabbits. This finding corresponds with the apparent ADF faecal digestibility in rabbits. This may be related to FR increasing the fibrinolytic activity (cellulase and xylanase activities) in rabbits [6].

FR might participate in stabilizing the intestinal microbial balance and thus inhibit pathogenic bacteria development in fattening rabbits [27]. Hence, FR can alter the diversity of the gut microbiota and reduce intestinal inflammatory responses in animals [51]. Zhuang et al. [30] demonstrated that FR improved immune function by regulating the secretion of interferon-γ, interleukin-10, and tumour necrosis factor-α. Notably, *Akkermansia* is involved in inflammation and anti-inflammatory effects, and colitis resulted from increased *Akkermansiaceae* abundance in mice [52]. In the present study, we found that FR increased caecal *Akkermansia* abundance, perhaps because FR resulted in increased antioxidant activity (e.g., T-AOC and SOD activities) and reduced oxidation products (e.g., MDA and ·OH) in rabbits.

Bacteria of the *Ruminococcaceae* family break down substances such as cellulose, proteins, and lipids, releasing short-chain fatty acids, amino acids, and lipid metabolites [53]. These metabolites not only provide energy and nutrients for intestinal epithelial cells but also increase the acidity of the intestinal wall, inhibit the growth of harmful bacteria, and maintain the balance of the intestinal microbiota [54]. Specifically, FR not only modulates the overall structure of the gut microbiota but also selectively enriches anti-inflammatory bacteria (e.g., *Oscillibacter*, *Allobaculum*, and *Lachnospiraceae*_NK4A136_group) and decreases the abundances of proinflammatory pathogenic bacteria (e.g., *Bifidobacterium*, *Bacteroides*, and *Lachnoclostridium*) [4]. Thus, FR might regulate the gut microbiota balance and then improve immune response in rabbits [27]. Additionally, *Ruminococcus* is a genus of bacteria that produces abundant propionic and butyric acids and can participate in feed digestion and maintain intestinal barrier function [55]. The results of this study revealed that FR increased the abundance of *Ruminococcus* in the caecal content, indicating that FR might improve nutrient digestibility, reduce the inflammatory response, and improve antioxidant activity in rabbits. This observation suggested that FR improved nutrient utilisation and plasma antioxidant activity in rabbits. Consistent with our results, Combes et al. [31] reported that FR regulated caecal microbiota contents, especially those of dominant genera belonging to the *Ruminococcaceae* family, in young rabbits.

β-alanine is a type of β-amino acid that exists in nature and does not participate in protein synthesis [56]; it is a metabolite of uracil and cytosine [57]. On the one hand, the function of β-alanine in animals is achieved mainly through the synthesis of carnosine, which is an endogenous active peptide that has the ability to scavenge oxidative free radicals and enhance antioxidant properties [58,59]. On the other hand, β-alanine can alleviate oxidative stress, inhibit fat oxidation, and thus enhance the body’s antioxidant capacity [60]. For example, β-alanine has strong antioxidant activity, which can increase the activity of SOD in muscle tissue and serum, reduce the MDA content, and increase the antioxidant capacity of muscles in animals [61]. Similarly, dietary supplementation with β-alanine could increase GSH-Px activity and reduce the MDA content in broiler chickens [62,63]. In this study, FR was found to enrich β-alanine metabolism in rabbits, possibly by enhancing antioxidant function (Table 6). In brief, FR can regulate microorganisms and metabolites in the caecal contents of rabbits, thereby increasing their antioxidant capacity.

## 5. Conclusions

In conclusion, FR increased the apparent faecal digestibility of acid detergent fibre, nitrogen digestibility, nitrogen retention, gross energy digestibility, and gross energy retention values. FR increased the total antioxidant capacity and superoxide dismutase activities and reduced the triglyceride, creatinine, high-density lipoprotein cholesterol, malondialdehyde, and hydroxyl free radical contents. FR increased *Firmicutes* and *Ruminococcus* abundances in the caecal content but decreased *Akkermansiaceae* abundance. FR simultaneously changed the structure of caecal metabolites in growing rabbits. These results indicated that FR can enhance nutrient utilisation, improve antioxidant activity, and alter caecal microbiota composition by increasing beneficial bacteria and modifying metabolic pathways in growing rabbits. However, we only tested 80% FR in this study, and further studies are needed to determine the impact of different levels of FR on bodily health in rabbits. Another potential limitation was that we did not compare other intestinal microbiota and metabolites in rabbits.

## Figures and Tables

**Table 1:** The ingredient of basal diet.

Ingredient, g/kg	Content
Corn	237
Soybean meal	67
Rapeseed meal	32
Cottonseed meal	31
Wheat bran	158
Alfalfa	425
CaHPO_4_	4.0
NaCl	3.5
Stone powder	2.5
Compound premix	40

Compound premix contained per kg: vitamin D_3_, 1000 IU; vitamin A, 15,000 IU; vitamin E, 300 IU; vitamin B_3_, 300 mg; vitamin K, 20 mg; calcium pantothenate, 200 mg; folic acid, 4 mg; I, 3.5 mg; Fe, 1500 mg; Mn, 500 mg; Cu, 300 mg; Zn, 1500 mg; biotin, 1 mg.

**Table 2:** The chemical composition of basal diet.

Chemical Composition	Content
Dry matter, g/kg	894
Crude protein, g/kg of DM	163
Gross energy, MJ/kg of DM	15.2
Neutral detergent fibre, g/kg of DM	348
Acid detergent fibre, g/kg of DM	214
Ash, g/kg of DM	97

Values represent the means of three replicates (*n* = 3). DM, dry matter.

**Table 3:** Effect of feed restriction on dry matter intake, growth performance, and apparent faecal digestibility of growing rabbits.

Item	AL	FR	SEM	*p*-Value
Dry matter intake, g/d	117.25 ^a^	93.39 ^b^	0.802	<0.001
Initial weight, g	1866.58	1877.67	53.384	0.885
Final weight, g	2693.53	2681.86	51.211	0.873
Average daily weight gain, g	19.69	19.15	1.794	0.833
Feed conversion ratio	6.18	5.67	0.522	0.499
Apparent faecal digestibility, %				
Dry matter	35.67	34.66	1.321	0.617
Neutral detergent fibre	29.64	29.37	1.471	0.904
Acid detergent fibre	18.57 ^b^	20.88 ^a^	0.514	0.034
Ash	25.23	25.02	0.631	0.826

^a,b^ Means with different superscripts within a row differ significantly (*p* < 0.05). Values represent the means of twelve replicates (*n* = 12). SEM, standard error of the mean. AL, rabbit was fed *ad libitum* diet; FR, rabbit was fed 80% *ad libitum* diet. The same as below.

**Table 4:** Effect of feed restriction on nitrogen utilisation of growing rabbits.

Item	AL	FR	SEM	*p*-Value
Nitrogen intake, g/d	3.07 ^a^	2.44 ^b^	0.016	<0.001
Nitrogen excretion in faeces, g/d	0.74 ^a^	0.49 ^b^	0.013	0.002
Nitrogen excretion in urine, g/d	0.05 ^a^	0.02 ^b^	0.002	0.001
Total nitrogen excretion, g/d	0.79 ^a^	0.51 ^b^	0.015	0.002
Nitrogen digestibility, %	75.95 ^b^	79.92 ^a^	0.433	0.003
Nitrogen retention, %	74.45 ^b^	78.96 ^a^	0.934	0.027

^a,b^ Means with different superscripts within a row differ significantly (*p* < 0.05). Values represent the means of twelve replicates (*n* = 12). SEM, standard error of the mean. AL, rabbit was fed *ad libitum* diet; FR, rabbit was fed 80% *ad libitum* diet. Nitrogen retention (%) = [(nitrogen intake-total nitrogen excretion)/nitrogen intake] × 100.

**Table 5:** Effect of feed restriction on energy utilisation of growing rabbits.

Item	AL	FR	SEM	*p*-Value
Gross energy intake, kJ/d	1784.55 ^a^	1421.45 ^b^	9.236	<0.001
Gross energy excretion in faeces, kJ/d	918.11 ^a^	614.10 ^b^	4.880	<0.001
Gross energy excretion in urine, kJ/d	23.35 ^a^	9.88 ^b^	0.502	<0.001
Total gross energy excretion, kJ/d	941.46 ^a^	623.98 ^b^	5.139	<0.001
Gross energy digestibility, %	48.55 ^b^	56.80 ^a^	0.304	<0.001
Gross energy retention, %	47.24 ^b^	56.10 ^a^	0.311	<0.001

^a,b^ Means with different superscripts within a row differ significantly (*p* < 0.05). Values represent the means of twelve replicates (*n* = 12). SEM, standard error of the mean. AL, rabbit was fed *ad libitum* diet; FR, rabbit was fed 80% *ad libitum* diet. Gross energy retention (%) = [(gross energy intake-total gross energy excretion)/gross energy intake] × 100.

**Table 6:** Effect of feed restriction on blood biochemical, lipid metabolism, and antioxidant activity parameters in rabbit.

Item	AL	FR	SEM	*p*-Value
Biochemical parameters				
Glucose, mmol/L	5.71	5.19	0.190	0.069
Total protein, g/L	3.56	3.49	0.050	0.320
Albumin, g/L	62.54	62.01	3.081	0.904
Lipid metabolism				
Triglyceride, mmol/L	0.77 ^a^	0.57 ^b^	0.063	0.042
Creatinine, μmol/L	118.87 ^a^	97.53 ^b^	5.161	0.008
Total cholesterol, mmol/L	2.86	2.86	0.402	0.995
Low-density lipoprotein cholesterol, mmol/L	0.75	0.57	0.073	0.091
High-density lipoprotein cholesterol, mmol/L	1.30 ^a^	1.02 ^b^	0.081	0.022
Antioxidant activity				
Total antioxidant capacity, U/mL	0.76 ^b^	1.15 ^a^	0.129	0.048
Superoxide dismutase, U/mL	106.56 ^b^	136.66 ^a^	0.532	<0.001
G lutathione peroxidase, U/mL	23.68	23.08	1.455	0.776
Catalase, U/mL	2.82	2.16	0.407	0.281
2,2-diphenyl-1-picrylhydrazyl scavenging activity, %	81.42	71.70	4.107	0.108
Malondialdehyde, nmol/mL	0.89 ^a^	0.70 ^b^	0.016	0.017
Hydroxyl free radical, U/mL	1341 ^a^	1150 ^b^	28.450	<0.001
Superoxide anion, U/L	334.06	333.67	4.264	0.949

^a,b^ Means with different superscripts within a row differ significantly (*p* < 0.05). Values represent the means of twelve replicates (n = 12). SEM, standard error of the mean. AL, rabbit was fed ad libitum diet; FR, rabbit was fed 80% ad libitum diet.

## Data Availability

The caecal microbiota datasets presented can be found in online repositories, and the BioProject ID is PRJNA1222279.
